# Longitudinal associations between generative artificial intelligence adoption and university PE teachers’ professional competence

**DOI:** 10.3389/fpsyg.2026.1775028

**Published:** 2026-03-05

**Authors:** Gang Zhou, Qishun Yang, Xiao Chen

**Affiliations:** 1 School of Football, Wuhan Sports University, Wuhan, Hubei, China; 2 School of Physical Education, Wuhan Business University, Wuhan, Hubei, China; 3 School of Athletic Training, Tianjin University of Sport, Tianjin, China; 4 Department of Physical Education, Zhongnan University of Economics and Law, Wuhan, Hubei, China

**Keywords:** cross-laggedpanel model, generative artificial intelligence adoption, self-regulated learning, teaching experience, university physical education teachers

## Abstract

**Background:**

Generative artificial intelligence (GenAI) is increasingly integrated into educational practice, yet longitudinal evidence regarding its role in teachers’ professional development remains limited, particularly in physical education contexts characterized by embodied teaching.

**Methods:**

This study employed a three-wave longitudinal design over 1 year to examine the dynamic associations between GenAI adoption and professional competence among 558 university physical education teachers in China. Cross-lagged panel modeling was used to test the longitudinal mediating role of self-regulated learning and the moderating effect of teaching experience.

**Results:**

The results showed that GenAI adoption exhibited a significant and sustained positive association with teachers’ professional competence across time. Self-regulated learning functioned as a longitudinal mediator linking GenAI adoption to professional competence. Moreover, teaching experience negatively moderated this indirect pathway, such that the mediation effect was stronger among early-career teachers than among those with longer teaching experience.

**Conclusion:**

By integrating the Technological Pedagogical Content Knowledge (TPACK) framework with self-regulated learning and career stage perspectives, this study extends prior cross-sectional research by elucidating the dynamic mechanisms through which GenAI adoption is associated with teachers’ professional competence development. The findings highlight the importance of differentiated and targeted digital empowerment strategies in physical education.

## Introduction

Teachers constitute the cornerstone of building a strong education system, and enhancing their professional competence has become a central issue in contemporary educational reform. Since the 18th National Congress of the Communist Party of China, the Chinese government has continuously advanced the development of the teaching workforce by issuing a series of landmark policy documents, including Opinions on Comprehensively Deepening the Reform of Teacher Workforce Development in the New Era and Opinions on Promoting the Spirit of Educators and Strengthening the Development of a High-Quality and Professional Teaching Workforce in the New Era. These documents explicitly identify the enhancement of professional competence as a strategic priority for the high-quality development of teachers (Luo and Dong, 2025). In 2025, the Notice of the General Office of the Ministry of Education on Organizing and Implementing the Digital Empowerment for Teacher Development Initiative further emphasized the role of digital empowerment in facilitating teachers’ proactive adaptation to technological transformation (General Office of the Ministry of Education, 2025). Against this backdrop, generative artificial intelligence (GenAI) has emerged as a key driver of educational transformation (Giannakos et al., 2025). This policy orientation suggests that GenAI, as a prominent manifestation of large-scale model technologies, has been systematically embedded within China’s educational reform framework. Unlike previous generations of educational technology, GenAI is characterized by its adaptive and responsive support functions, which make it especially conducive to sustaining teachers’ ongoing professional development (Li et al., 2025). Therefore, investigating the mechanisms through which artificial intelligence influences teachers is of critical theoretical and practical significance.

Powered by deep learning and large-scale data training, generative artificial intelligence substantially enhances users’ learning efficiency through human-like interaction patterns (Wang et al., 2025). In the context of higher education in physical education, teachers’ professional competence encompasses four core dimensions: motor skill instruction, instructional design and implementation, research and innovation capacity, and continuous self-development. Existing studies suggest that GenAI can provide immediate error correction through action recognition and three-dimensional demonstrations, optimize classroom instruction via data-driven precision support, and enhance research capability and autonomous learning through text generation functions (Wang and Wang, 2024; Hu and Min, 2025). Cross-sectional evidence has further demonstrated that GenAI adoption positively predicts professional competence among university teachers. Nevertheless, some studies caution that excessive reliance on GenAI may undermine teachers’ critical thinking and creativity (Ahmad et al., 2023). Given these mixed findings and the lack of longitudinal evidence, systematic tracking studies are needed to examine the dynamic mechanisms underlying these effects.

Although the application of generative artificial intelligence in education has expanded rapidly, its internal mechanisms of action remain insufficiently explained. As an active regulatory process encompassing cognitive monitoring, motivational maintenance, and behavioral adjustment, self-regulated learning (SRL) may serve as a key mediator between technology adoption and the enhancement of teachers’ professional competence (Wolters, 2003). Prior research indicates that the immediate interaction and feedback provided by GenAI can effectively promote learners’ self-regulated learning capacity (Lai and Jin, 2021), which in turn is closely associated with critical indicators of teacher competence, such as research productivity and professional development (Jud et al., 2024). However, empirical evidence regarding the specific mediating pathways of SRL in the relationship between technology adoption and professional competence remains limited. Moreover, teaching experience, as an important individual characteristic, may moderate the effects of GenAI adoption. As teaching experience increases, established instructional schemas tend to become more stable or even rigid, while teachers’ willingness and capacity to adopt digital technologies may decline (Song and Liu, 2024). Existing empirical investigations in this area have relied predominantly on regional or localized samples, and systematic validation based on nationally representative data is still lacking. In addition, most prior studies have employed cross-sectional or single-wave designs (Alwakid et al., 2025; Hava and Babayiğit, 2025), which limits the ability to capture temporal dynamics and infer causal mechanisms. Notably, the specific context of university physical education teachers has received relatively little scholarly attention (Gong et al., 2023).

To address these limitations, the present study draws on a three-wave longitudinal dataset collected from university physical education teachers in China during a period of accelerated digital transformation. This design allows for an examination of the developmental relationships between GenAI adoption and professional competence over time. By applying cross-lagged panel modeling and incorporating self-regulated learning as a longitudinal mediator and teaching experience as a moderator, this study seeks to elucidate the dynamic mechanisms and boundary conditions through which GenAI adoption contributes to teachers’ professional development.

## Literature review

### Generative artificial intelligence and teachers’ professional competence

GenAI, with its advantages in content generation and immediate feedback, has demonstrated considerable potential in enhancing teachers’ professional competence. The Technological Pedagogical Content Knowledge (TPACK) framework integrates Technological Knowledge (TK), Pedagogical Knowledge (PK), and Content Knowledge (CK), providing a conceptual foundation for understanding the deep integration of technology and teaching (Oved and Alt, 2025). In PE contexts, the embodied nature of motor skills requires teachers not only to convey theoretical principles but also to demonstrate and correct movement execution. This dual transformation between bodily experience and technological mediation substantially increases the complexity of technology–pedagogy integration.

Within this context, GenAI may facilitate the formation and advancement of PE teachers’ professional competence across three levels. First, at the level of knowledge construction, content generation, intelligent retrieval, and multimodal presentation enable the rapid expansion of teachers’ TK and CK while effectively reducing cognitive load (Feng, 2025). Second, at the level of instructional practice, real-time feedback mechanisms support teachers in dynamically adjusting instructional strategies, thereby reconstructing PK and fostering a more robust integrated TPACK structure (Bukar et al., 2024). Third, at the level of competence transformation, the deep embedding of GenAI into key processes such as curriculum design, motor performance analysis, and research innovation contributes to the systematic enhancement of professional competence. Experimental evidence provides preliminary support for these effects: pre-service teachers receiving GenAI-supported instruction demonstrated significantly higher levels of self-efficacy and higher-order thinking skills than those in control groups (Lu et al., 2024). Accordingly, the following hypothesis is proposed:

*H1*: GenAI adoption positively predicts professional competence among university PE teachers.

### The longitudinal mediating role of self-regulated learning

Self-regulated learning (SRL) refers to individuals’ capacity to actively regulate their cognition, motivation, and behavior across the phases of goal setting, strategy implementation, and outcome reflection (Zimmerman, 2000). Some scholars conceptualize SRL as a cyclical process comprising three stages: forethought, performance, and reflection (Wu et al., 2024). Within the relationship between GenAI adoption and professional competence, SRL functions as a bidirectional bridging mechanism.

GenAI provides differentiated support across the three stages of SRL. During the forethought stage, AI stimulates learning motivation through personalized interaction and contextualized questioning, thereby assisting teachers in clarifying learning goals. During the performance stage, AI-generated innovative solutions and diversified resources help teachers overcome cognitive challenges. During the reflection stage, targeted feedback enables teachers to identify problems and optimize learning strategies (Pintrich, 2000). Empirical studies indicate that, compared with conventional AI tools, GenAI more effectively enhances learners’ SRL through personalized feedback (*p* ≤ 0.05) (Ng et al., 2024).

Conversely, improvements in SRL contribute directly to the enhancement of teachers’ professional competence. A study involving 106 in-service teachers demonstrated that SRL enables educators to effectively manage their cognition, emotions, and motivation, thereby promoting professional development (Karlen et al., 2020). In PE instruction, where teachers must continuously adapt strategies within dynamic teaching environments and balance skill demonstration with theoretical explanation, SRL serves as a critical mechanism linking technological tools with pedagogical practice. Based on the above reasoning, the following hypothesis is proposed:

*H2*: SRL longitudinally mediates the relationship between GenAI adoption and teachers’ professional competence.

### The moderating role of teaching experience

Teaching experience, as a temporal marker of professional development, may moderate the effects of technology adoption. Career stage theory conceptualizes teacher development as progressing through stages of growth, maturity, and maintenance, with systematic differences in technology acceptance and learning strategies across stages (Luo et al., 2008).

A defining characteristic of PE instruction lies in its reliance on embodied cognition. Embodied cognition theory posits that cognitive processes are deeply rooted in interactions between the body and the environment (Farina, 2021). For PE teachers, instructional knowledge is largely stored in the form of bodily schemas—implicit motor programs developed through prolonged physical practice. While teachers with greater teaching experience accumulate rich embodied instructional expertise, a “cognition–action transformation load” may arise when integrating AI-generated textual or visual recommendations into embodied teaching practice. Specifically, translating digital suggestions into concrete motor instruction requires additional cognitive resources to reconcile discrepancies between established bodily experience and digitally mediated information.

Empirical evidence supports this reasoning. Early-career teachers, characterized by higher cognitive flexibility and stronger digital-native traits, exhibit significantly greater willingness to adopt technology than their more experienced counterparts (Xu and Meyer, 2007). In PE contexts, teachers with fewer years of experience are more likely to incorporate AI tools as cognitive scaffolds in instruction, whereas teachers with longer teaching experience may encounter technology adaptation barriers due to path dependence. Accordingly, the following hypothesis is proposed:

*H3*: Teaching experience negatively moderates the indirect pathway through which GenAI adoption influences professional competence via SRL, such that the strength of this pathway decreases as teaching experience increases.

## Participants and methods

### Study design and participants

This study employed a three-wave panel survey design to investigate the relationships between generative AI acceptance, self-regulated learning, and professional competence among physical education teachers. Unlike experimental designs that involve manipulating variables and measuring their effects, our approach utilized a structured survey methodology to capture naturally occurring attitudes and perceptions over time (Blasi et al., 2001). First, all measurement instruments underwent rigorous validation procedures. Content experts in statistics and physical education reviewed the instruments to ensure scientific rigor and content validity. A pilot test was conducted in August 2024 with 30 university PE teachers, resulting in two key refinements: (a) redundant items were merged or removed to limit completion time to approximately 12 min, and (b) semantic ambiguities were corrected to enhance readability and applicability among Chinese university PE teachers. The research protocol received approval from the Ethics Committee of Tianjin University of Sport (Approval No. TJUS2025-085).

Electronic questionnaires were distributed systematically to the target sample via the Wenjuanxing platform. Following established methodological guidelines for longitudinal survey research (Grimm et al., 2021), data collection occurred at three distinct time points: July 2024 (T1), January 2025 (T2), and July 2025 (T3), each separated by a six-month interval. This three-wave design is consistent with contemporary longitudinal survey methodology, allowing for the systematic examination of time-lagged effects and temporal relationships among key variables over the one-year study period.

Regarding data management, 630 questionnaires were distributed at T1, yielding 617 valid responses (97.9%). At T2 and T3, 602 (97.6%) and 587 (97.5%) valid questionnaires were obtained, respectively. Through anonymous coding and verification of contact information, a final analytical sample of 558 teachers was successfully matched across all three waves (representing 90.4% of the baseline sample) and used for all subsequent statistical analyses and demographic reporting.

Attrition was attributable to sick leave or professional training (4.1%), job transfer or reassignment (2.7%), and invalid responses (2.8%). The overall attrition rate was 9.6%, well below the commonly accepted bias threshold of 20% for longitudinal studies (Cummings, 2013). To ensure internal validity and consistency across the longitudinal cross-lagged panel model, only participants with complete data for all three time points N = 558 were included in the final analysis, as reflected in Table 1.

**Table 1:** Demographic characteristics of the participants.

Dimension	Category	*n*	%
Gender	Male	388	69.5%
	Female	170	30.5%
Educational level	Bachelor	168	30.1%
	Master	340	60.9%
	Doctor	50	9%
Academic rank	Professor	20	3.6%
	Associate professor	68	12.2%
	Lecturer	403	72.2%
Marital status	Married	398	71.3%
	Unmarried	160	28.7%
Work region	Central China	77	13.8%
	Eastern China	98	17.6%
	Southern China	89	15.9%
	Northern China	73	13.1%
	Southwestern China	91	16.3%
	Northwestern China	57	10.2%
	Northeastern China	73	13.1%
Teaching experience	0–10 years	179	32.1%
	10–20 years	196	35.1%
	≥ 20 years	183	32.8%

### Measures

#### Generative artificial intelligence acceptance inventory

GenAI adoption was assessed using the Generative Artificial Intelligence Acceptance Inventory developed by Yilmaz et al. (2024). It is important to note that this instrument assesses psychological acceptance and behavioral intentions toward AI rather than actual usage frequency or behavior logs. Based on the Technology Acceptance Model framework (Venkatesh and Davis, 2000), the scale comprises 20 items across four dimensions: performance expectancy, effort expectancy, facilitating conditions, and social influence. This approach is consistent with established methodologies for measuring technology adoption in educational settings, where acceptance precedes and predicts actual usage behavior. All items were rated on a five-point Likert scale (1 = strongly disagree, 5 = strongly agree).

Reliability and validity analyses demonstrated excellent psychometric properties. The overall Cronbach’s *α* coefficient was 0.97, with subscale α values ranging from 0.87 to 0.96. Confirmatory factor analysis (CFA) indicated good model fit (*χ*^2^ = 2.113, CFI = 0.97, GFI = 0.88, IFI = 0.97, TLI = 0.97, RMSEA = 0.067, SRMR = 0.033). In addition, convergent validity (AVE = 0.54–0.74) and composite reliability (CR = 0.77–0.93) met recommended psychometric criteria (Fornell and Larcker, 1981), indicating satisfactory measurement reliability and structural validity.

## Self-regulated learning questionnaire

SRL was measured using the Self-Regulated Learning Questionnaire developed by Oz and Sen (2018). The instrument consists of 39 items covering five dimensions: learning strategies, self-evaluation, help seeking, time management and planning, and information acquisition. All items were rated on a five-point Likert scale (1 = never, 5 = always).

Psychometric evaluation showed strong reliability and validity. The overall Cronbach’s α coefficient was 0.94. Exploratory factor analysis yielded a Kaiser–Meyer–Olkin (KMO) value of 0.945, and Bartlett’s test of sphericity was significant (*χ*^2^ = 7320.964, *p* < 0.01). The five-factor structure explained 44.95% of the total variance, with factor loadings ranging from 0.311 to 0.797. CFA further indicated good model fit (RMSEA = 0.035, SRMR = 0.047, CFI = 0.98, NFI = 0.94, TLI = 0.98, *χ*^2^/df = 1.50), supporting the scale’s reliability and structural validity.

## Physical education teachers’ professional competence scale

Teachers’ professional competence was assessed using the Professional Competence Scale developed by Akkermans et al. (2013). The scale includes 21 items across six dimensions: reflective motivation, reflective qualities, networking, self-profiling, work exploration, and career control, with each dimension comprising three to four items. Responses were recorded on a five-point Likert scale (1 = completely disagree, 5 = completely agree).

Reliability and validity analyses indicated satisfactory psychometric properties. The overall Cronbach’s *α* coefficient was 0.90, with subscale α values ranging from 0.76 to 0.82. CFA supported a first-order six-factor model with good fit indices (*χ*^2^(174) = 253.45, CFI = 0.96, TLI = 0.95, GFI = 0.91, RMSEA = 0.046). Discriminant validity was further confirmed (Δ*χ*^2^(16) = 365.97, *p* < 0.001; CFI = 0.96, TLI = 0.94, GFI = 0.93, RMSEA = 0.07), indicating that the scale is appropriate for assessing professional competence among PE teachers.

To enhance comparability across instruments, all three measures were administered using a consistent five-point Likert-type response format. Standardizing the response scale is recommended in survey and scale-design research because it helps reduce extraneous format-related variance and facilitates coherent integration of multiple measures within a single analytic framework (Krosnick and Fabrigar, 1997). Moreover, claims of metric comparability are most rigorously evaluated within the measurement invariance framework, which clarifies the conditions under which scale scores and model parameters are meaningfully comparable (Millsap, 2012).

### Statistical analysis

This study implemented a comprehensive analytical approach to examine the temporal relationships between variables across the three measurement waves. Data analysis proceeded through several sequential stages:

First, data preparation and preliminary analyses were conducted using SPSS 25.0. This included data cleaning procedures to identify and address missing values, outliers, and potential violations of statistical assumptions. Descriptive statistics (means, standard deviations, skewness, and kurtosis) were calculated to examine the distributional properties of all study variables at each time point. Correlation analyses were performed to assess bivariate relationships between variables both within and across measurement waves.

Second, measurement invariance testing was conducted to ensure that the measurement properties of the instruments remained consistent across the three time points, a critical prerequisite for valid longitudinal comparisons. This involved a series of increasingly constrained confirmatory factor analysis models to test configural, metric, and scalar invariance using Mplus 8.3.

Third, cross-lagged panel models (CLPMs) were constructed using Mplus 8.3 to examine the bidirectional relationships between variables over time. These models simultaneously estimated autoregressive paths (stability of constructs over time) and cross-lagged paths (influence of one variable at Time t on another variable at Time *t* + 1), while controlling for within-time correlations. To integrate instruments with varying lengths (20, 39, and 21 items), we utilized a dimension-level parceling approach (sub-dimension means) as observed indicators for the Cross-Lagged Panel Model (CLPM). This strategy standardizes the measurement framework, enhances model parsimony, and provides more stable parameter estimates compared to item-level modeling (Bandalos, 2002; Little et al., 2022).

Finally, longitudinal mediation and moderation analyses were conducted to test specific hypothesized mechanisms. Bootstrap procedures with 5,000 resamples were employed to generate bias-corrected confidence intervals for indirect effects. For moderation analyses, interaction terms were created and tested within the structural equation modeling framework.

## Results

### Common method bias test

To assess potential common method bias, Harman’s single-factor test was conducted separately for the three waves of questionnaire data. The results showed that five, six, and four factors with eigenvalues greater than 1 were extracted at T1, T2, and T3, respectively. The first factor accounted for 22.82%, 23.08%, and 19.17% of the total variance across the three waves, all of which were well below the recommended threshold of 40% (Podsakoff and Organ, 1986). These findings indicate that common method bias was not a serious concern in the present study.

### Descriptive statistics and correlation analysis

To systematically examine the basic association patterns among GenAI adoption, SRL, and professional competence (PC), Pearson correlation coefficients were calculated at three measurement points (T1, T2, and T3). The results are presented in Table 2.

**Table 2:** Descriptive statistics and correlations among study variables.

Variables	Mean±SD	Skewness	Kurtosis	T1 GenAI	T2 GenAI	T3 GenAI	T1 SRL	T2 SRL	T3 SRL	T1 PC	T2 PC	T3 PC
T1 GenAI	3.26 ± 0.77	0.125	−0.452	1								
T2 GenAI	3.52 ± 0.73	0.089	−0.311	0.353**	1							
T3 GenAI	3.69 ± 0.70	−0.142	0.258	0.330**	0.334**	1						
T1 SRL	3.28 ± 0.79	0.211	−0.518	0.439**	0.473**	0.331**	1					
T2 SRL	3.54 ± 0.76	0.104	−0.376	0.459**	0.444**	0.479**	0.501**	1				
T3 SRL	3.74 ± 0.68	−0.205	0.115	0.399**	0.414**	0.345**	0.444**	0.508**	1			
T1 PC	3.33 ± 0.81	0.312	−0.614	0.363**	0.390**	0.437**	0.443**	0.487**	0.400**	1		
T2 PC	3.51 ± 0.80	0.156	−0.289	0.404**	0.364**	0.298**	0.496**	0.487**	0.416**	0.453**	1	
T3 PC	3.79 ± 0.66	−0.318	0.736	0.415**	0.444**	0.419**	0.453**	0.477**	0.449**	0.428**	0.358**	1

*p < 0.05; **p < 0.01. GenAI, generative artificial intelligence adoption; SRL, self-regulated learning; PC, professional competence.

Descriptive statistics revealed a stable upward trend in the mean levels of all three variables over time. Specifically, GenAI adoption increased from 3.26 at T1 to 3.69 at T3; SRL increased from 3.28 to 3.74; and PC increased from 3.33 to 3.79. The standard deviations of the variables ranged from 0.66 to 0.79, indicating moderate dispersion alongside overall improvement. The absolute values of skewness ranged from 0.042 to 0.518, and the absolute values of kurtosis ranged from 0.115 to 0.736. These values fall well within the recommended criteria (Ryu, 2011; Kim, 2013), indicating that the data approximate a normal distribution and are suitable for maximum likelihood estimation in structural equation modeling.

Correlation analyses further showed that at baseline (T1), GenAI adoption was positively correlated with SRL (*r* = 0.439, *p* < 0.05) and PC (*r* = 0.363, *p* < 0.05), and SRL was positively correlated with PC (*r* = 0.443, *p* < 0.05). These correlation patterns were replicated at T2 and T3, suggesting good cross-temporal stability among the three variables.

Regarding temporal stability, the adjacent-wave stability correlations were 0.353 and 0.334, respectively. The corresponding coefficients for SRL were 0.501 and 0.508, and for PC were 0.453 and 0.449. All values were within a moderate range, ensuring sufficient temporal consistency while avoiding excessive stability that could interfere with subsequent cross-lagged analyses (Hamaker et al., 2015). In addition, the maximum correlation among variables in the correlation matrix was 0.508, below the critical threshold for multicollinearity, satisfying the assumptions for subsequent structural equation modeling.

Overall, GenAI adoption, SRL, and PC exhibited consistent, stable, and directionally positive correlations across all three measurement waves, meeting the theoretical requirements for longitudinal analysis (Cole and Maxwell, 2003) and providing a solid foundation for further model testing.

### Longitudinal mediation model analysis

Cross-lagged panel modeling was conducted using Mplus 8.3 to analyze the three-wave longitudinal data. The model simultaneously estimated autoregressive paths, cross-lagged effects among GenAI adoption, SRL, and PC, as well as the longitudinal mediating role of SRL and the moderating effect of teaching experience.

Because the model was a saturated mean-structure model based on dimension-level composite scores, chi-square statistics and degrees of freedom were not reported; instead, model evaluation focused on key fit indices. The results indicated satisfactory model fit, with CFI = 0.954 and SRMR = 0.040, both meeting recommended criteria (Hu and Bentler, 1999). Although TLI was 0.810, falling below the conventional cutoff of 0.90, it remains acceptable in complex longitudinal mean-structure models (Marsh et al., 2004) Overall, the model demonstrated adequate fit, providing a solid basis for the examination of path coefficients and underlying mechanisms.

### Path analysis

**Figure 1:**
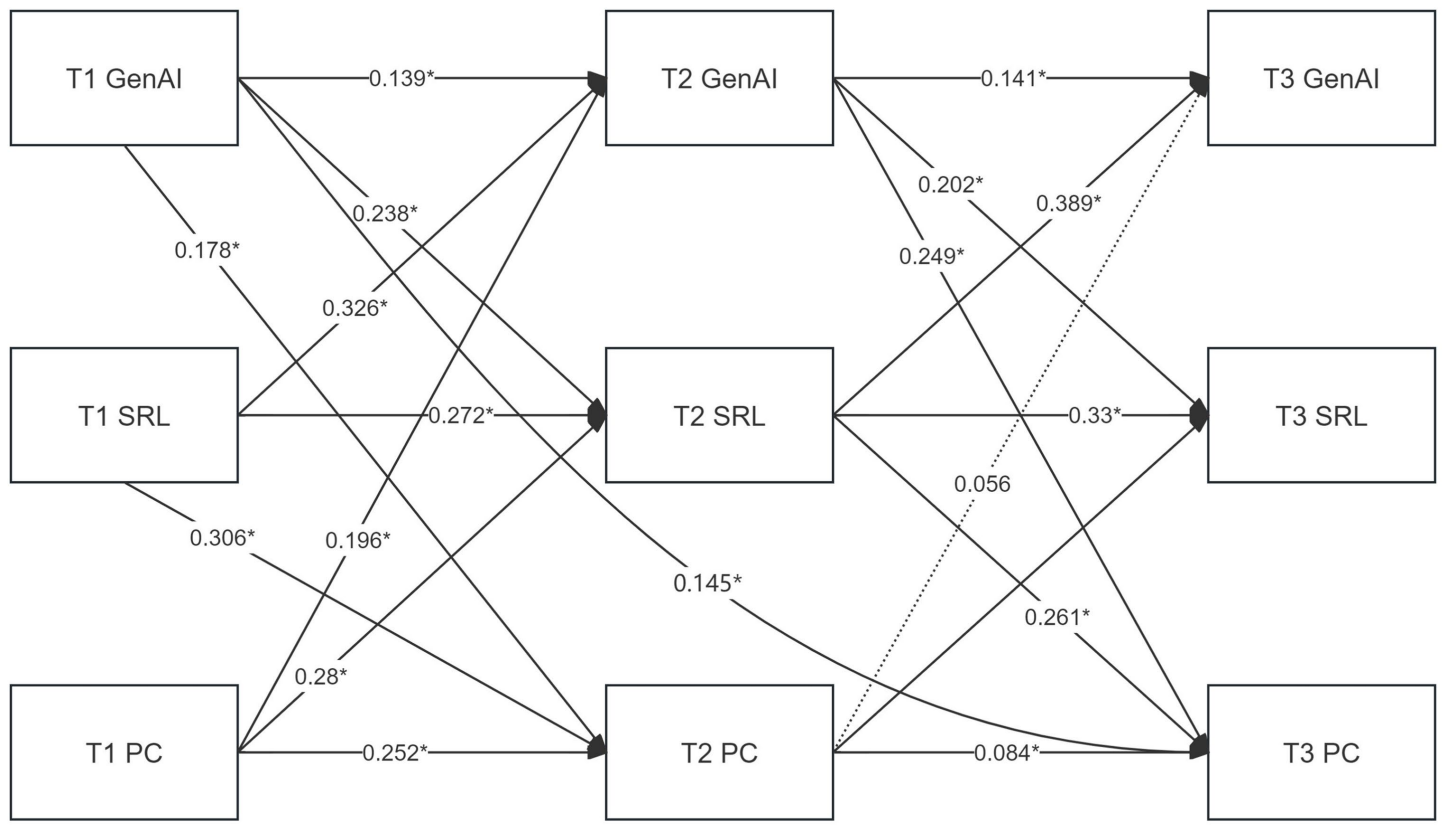
Cross-lagged panel model. *p<0.05; GenAI, generative artificial intelligence adoption; SRL, self-regulated learning; PC, professional competence.

Figure 1 presents the temporal pathways among variables within the three-wave cross-lagged panel model. First, GenAI adoption exhibited a significant and sustained direct effect on PC. Specifically, GenAI adoption at T1 positively predicted PC at T2 (*β* = 0.178, *p* < 0.05), and the direct effect from T2 to T3 was also significant (*β* = 0.249, p < 0.05). In addition, the cross-time effect of T1 GenAI adoption on T3 PC reached statistical significance (*β* = 0.145, *p* < 0.05), indicating both short-term and enduring effects of GenAI adoption on professional competence.

Second, the positive influence of GenAI adoption on SRL was also robust. GenAI adoption at T1 significantly predicted SRL at T2 (*β* = 0.238, *p* < 0.05), and the predictive effect from T2 to T3 remained significant (*β* = 0.202, *p* < 0.05), suggesting that continuous use of GenAI tools contributes to the progressive optimization of teachers’ self-regulated learning strategies.

Third, the positive role of SRL in enhancing PC was strongly supported. SRL at T1 significantly predicted PC at T2 (*β* = 0.306, *p* < 0.05), and the effect from T2 to T3 was also significant (*β* = 0.261, *p* < 0.05), underscoring the critical mediating role of SRL in the development of teachers’ professional competence.

Taken together, the path coefficients supported a dynamic mediation mechanism of “GenAI adoption → SRL → PC.” GenAI adoption not only enhanced professional competence through direct pathways but also exerted progressive indirect effects by strengthening SRL, revealing the multi-level role of technology adoption in promoting teachers’ professional development.

### Moderated mediation analysis

To examine the moderating role of teaching experience within the mediation pathway, a moderated mediation analysis was conducted following the procedure proposed by Edwards and Lambert (Edwards and Lambert, 2007). This analysis systematically investigated the role of teaching experience in the “GenAI adoption → SRL → PC” chain (see Table 3).

**Table 3:** Regression results of the moderated mediation model.

Outcome variable	Predictor	*R*	*R* ^2^	*f*	Effect	*t*
T2 SRL	T1 GenAI	0.471	0.222	52.657	0.086	0.601
	Teaching experience				−0.508	−2.224*
	T1 GenAI * teaching experience				0.171	2.5*
T3 PC	T1 GenAI	0.525	0.276	105.77	0.214	6.104***
	T2 SRL				0.315	8.929***

*p < 0.05; ***p < 0.001. GenAI, generative artificial intelligence adoption; SRL, self-regulated learning; PC, professional competence.

First, path analysis results showed that in the model with T2 SRL as the outcome variable, the main effect of T1 GenAI adoption was not significant (*β* = 0.086, *t* = 0.601, *p* > 0.05), whereas the main effect of teaching experience was significantly negative (*β* = −0.508, *t* = −2.224, *p* < 0.05). More importantly, the interaction between T1 GenAI adoption and teaching experience was significant (*β* = 0.171, *t* = 2.500, *p* < 0.05), indicating that teaching experience significantly moderated the effect of GenAI adoption on SRL. The model explained 22.2% of the variance in SRL (*R*^2^ = 0.222) and demonstrated good overall fit (*F* = 52.657, *p* < 0.05).

In the model with T3 PC as the outcome variable, T1 GenAI adoption positively predicted PC (*β* = 0.214, *t* = 6.104, *p* < 0.05), and T2 SRL also had a significant positive effect on T3 PC (*β* = 0.315, *t* = 8.929, *p* < 0.05). This model accounted for 27.6% of the variance in PC (*R*^2^ = 0.276, *F* = 105.77, *p* < 0.05), indicating that SRL partially mediated the relationship between GenAI adoption and professional competence.

**Figure 2:**
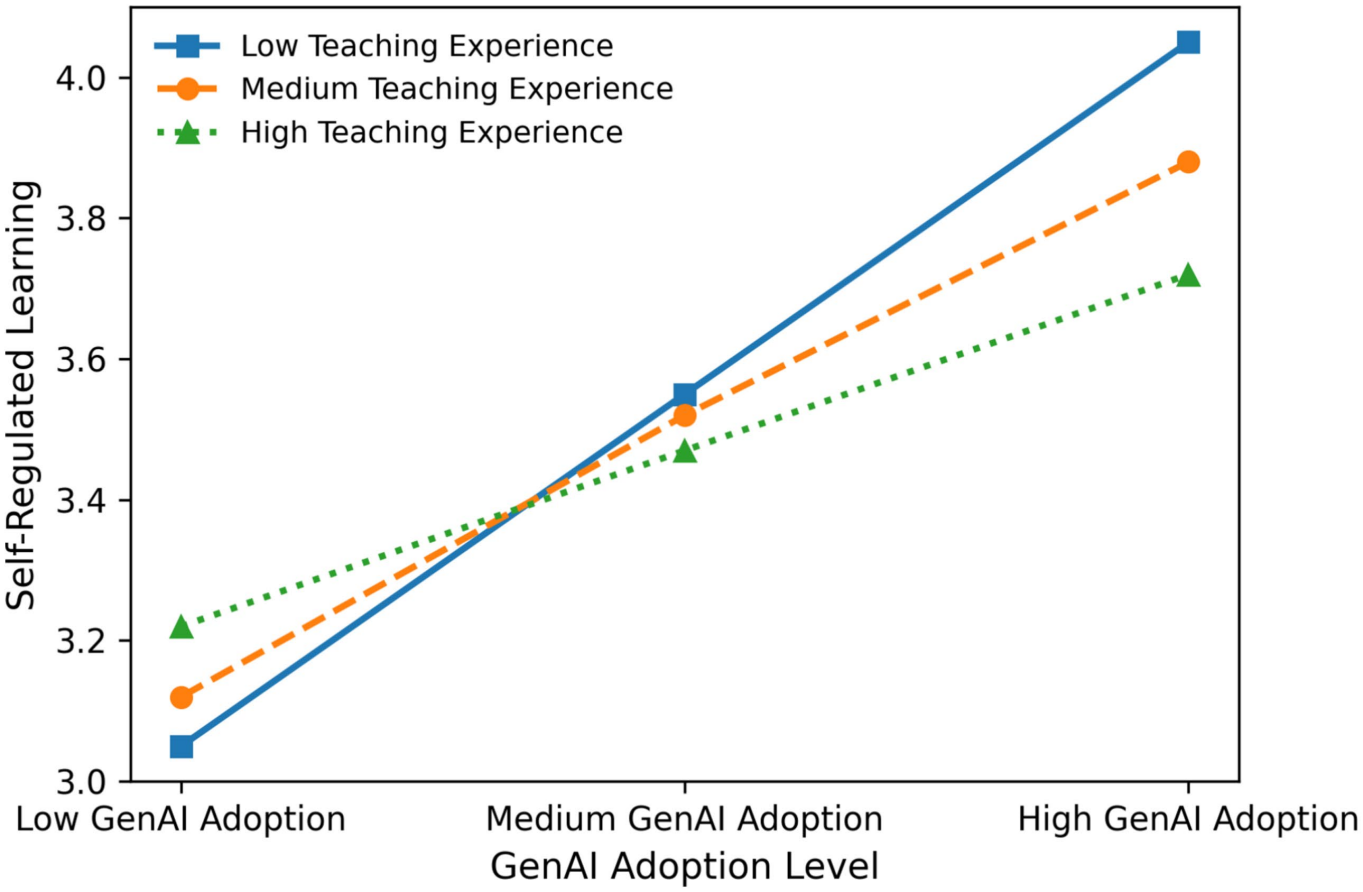
Moderating effect of teaching experience on the relationship between GenAI adoption and self-regulated learning.

Next, the sample was divided into three groups based on teaching experience: low (≤10 years), medium (11–20 years), and high (≥21 years). Conditional indirect effects were examined for each group. The results showed that the indirect effect was 0.186 for the low-experience group (Boot SE = 0.031, 95% CI [0.129, 0.251]), 0.137 for the medium-experience group (Boot SE = 0.019, 95% CI [0.101, 0.176]), and 0.089 for the high-experience group (Boot SE = 0.025, 95% CI [0.040, 0.141]). None of the confidence intervals included zero, indicating that the mediation effect was significant across all experience levels, while the magnitude of the indirect effect decreased as teaching experience increased (see Figure 2).

Finally, the index of moderated mediation was −0.049 (Boot SE = 0.021, 95% CI [−0.092, −0.009]), which was significantly different from zero, providing further evidence that teaching experience negatively moderated the strength of the mediation effect.

Overall, although the direct effect of GenAI adoption on SRL became nonsignificant after the interaction with teaching experience was introduced, the significant interaction indicates that this effect was contingent upon teaching experience. Teachers with fewer years of experience benefited more from GenAI adoption in terms of enhancing SRL, which in turn translated into higher levels of professional competence, whereas teachers with longer teaching experience derived comparatively weaker benefits along this pathway. Teaching experience thus not only moderated the effect of GenAI adoption on SRL but also indirectly shaped the ultimate impact of technology adoption on professional competence.

## Discussion

Using a three-wave longitudinal design, the present study tracked 558 university PE teachers over a one-year period to systematically examine the dynamic mechanisms through which GenAI adoption contributes to teachers’ professional development. The high retention rate (90.4%) ensured the robustness of the findings. The results revealed a serial pathway of “GenAI adoption → SRL → professional competence,” as well as a negative moderating effect of teaching experience, thereby providing a novel theoretical perspective on the digital development of PE teachers.

### GenAI adoption and professional competence of university PE teachers

This study systematically examined the mechanisms through which GenAI adoption enhances professional competence among university PE teachers. The results indicate that GenAI not only significantly promotes teachers’ professional competence, but that this effect also exhibits temporal accumulation. These findings provide strong support for Hypothesis 1 and are consistent with prior cross-sectional evidence reported by Tang et al. (2025). Unlike previous studies that focused primarily on single-time-point associations, the present three-wave design further substantiates a directional relationship between GenAI adoption and professional competence. Specifically, the path coefficient increased from 0.178 (T1 → T2) to 0.249 (T2 → T3), highlighting the cumulative nature of technology empowerment.

The cumulative effect of GenAI adoption can be understood as a developmental trajectory characterized by “progressive integration and spiral growth.” From the perspective of the TPACK framework, GenAI facilitates professional competence by dynamically restructuring the relationships among its constituent components. In the initial stage, AI tools expand teachers’ TK and CK by enabling immediate access to theoretical knowledge, particularly in areas such as biomechanics and exercise physiology (Lee and Moore, 2024). In the second stage, AI-supported instructional practice updates PK by fostering a pattern of data-driven pedagogical decision-making. In the third stage, these knowledge elements are integrated into TPACK (Granić, 2023), enabling teachers to flexibly design individualized training programs and implement precision teaching aligned with students’ diverse needs.

An important contribution of this study is the finding that the role of GenAI extends beyond instrumental support. According to the dynamic developmental view of TPACK, deep integration between TK and pedagogical content knowledge can give rise to new cognitive structures (Oved and Alt, 2025). Through sustained engagement with AI tools, teachers develop novel modes of data interpretation and model-based reasoning. This cognitive reconstruction suggests that technology not only transforms instructional practices but also reshapes teachers’ professional thinking, facilitating a transition from mere technology adoption to genuine competence development.

### The longitudinal mediating role of self-regulated learning

Based on three-wave longitudinal data, this study systematically examined the dynamic mediating role of SRL in the relationship between GenAI adoption and professional competence. The results demonstrate that SRL exerts a significant indirect effect, and that this effect strengthens over time. These findings provide robust support for Hypothesis 2 and are highly consistent with previous research (Zhou et al., 2024).

The mediating role of SRL follows a three-stage pattern of “activation–reinforcement–transformation.” During the activation stage, the interactive features of GenAI stimulate teachers’ metacognitive awareness, prompting them to clarify learning goals and formulate strategies (Zimmerman, 2000). In the reinforcement stage, AI-generated immediate feedback enhances teachers’ self-monitoring capacity, enabling real-time adjustment of learning pathways. In the transformation stage, accumulated self-regulatory experiences are consolidated into stable gains in professional competence. By extending Pintrich’s (Pintrich, 2000) SRL model into a longitudinal framework, this study demonstrates that sustained technological stimulation can produce a “compounding effect” of regulatory capacity.

Distinct from teachers in theory-oriented disciplines who primarily regulate cognitive strategies, PE teachers face a “triple regulation” challenge involving cognitive regulation, motor regulation, and situational regulation. GenAI provides multimodal regulatory support, which helps explain its distinctive empowering effect for PE teachers. Whereas prior studies often conceptualized SRL as a relatively stable trait, the present findings reveal its dynamic malleability, with mean levels increasing from 3.28 at T1 to 3.74 at T3. The longitudinal design captures the evolving nature of the mediating process, characterized by progressive activation, reinforcement, and transformation. These results suggest that teacher training programs should cultivate SRL awareness in early stages and gradually provide greater autonomy in later stages, facilitating a shift from externally regulated learning to self-regulated learning.

### The moderating role of teaching experience

The present study found that teaching experience exerted a significant negative moderating effect on the “GenAI adoption → SRL → professional competence” pathway (Index = −0.049, *p* < 0.05). The indirect effect was strongest among teachers with low teaching experience (≤10 years; 0.186), followed by those with medium experience (11–20 years; 0.137), and weakest among those with high experience (≥21 years; 0.089), indicating a clear declining trend. This “experience threshold” effect not only supports Hypothesis 3 but also highlights generational differences in technology empowerment within the PE context.

Dreyfus (2004) argued that expert teachers’ knowledge is largely embodied in the form of implicit bodily schemas, namely procedural motor experience. For example, when AI recommends that “the hip abduction angle should be 45 degrees,” novice teachers may directly use this numerical indicator as an instructional reference, whereas experienced teachers must perform complex transformations between digital information and embodied experience, resulting in a “cognition–embodiment transformation load” that weakens the positive effects of technology adoption. Moreover, this study reveals a paradox: greater teaching experience may correspond to lower effectiveness of technology integration. As Berman et al. (2002) noted, sport skills involve substantial tacit knowledge that is difficult to verbalize. Veteran coaches may intuitively detect subtle movement errors, yet such embodied judgments are not easily translated into parameters that AI systems can process. Consequently, deep experiential knowledge may act as a barrier to accepting AI-generated recommendations, as experienced teachers tend to rely more heavily on their embodied expertise.

It is noteworthy that the linear negative relationship observed in this study differs from findings in Western contexts. A meta-analysis by Tondeur et al. (2017) reported an inverted U-shaped relationship between teaching experience and technology integration, with teachers of medium experience demonstrating the highest levels of integration. In contrast, the present study identified early-career teachers as the most responsive group. This discrepancy may reflect contextual differences in disciplinary characteristics, teacher training systems, and the stage of digital transformation in higher education.

### Limitations and future directions

Despite its contributions, this study has several limitations. First, all core variables were measured using self-reported questionnaires. Future research could integrate classroom observations, learning platform log data, and student evaluations to construct a multi-source, asynchronous data framework, thereby enhancing measurement reliability and validity. Second, the representativeness of the sample may be limited. Although participants were drawn from seven major regions across eastern, central, and western China, teachers from “Double First-Class” universities were overrepresented, which may constrain the generalizability of the findings to private institutions and vocational colleges. Future studies could adopt stratified quota sampling and conduct cross-regional comparisons to examine whether socioeconomic differences moderate the proposed pathways.

### Practical implications and recommendations

Grounded in the TPACK framework, self-regulated learning theory, and career stage theory, the present study elucidates a longitudinal serial mechanism linking GenAI adoption, SRL, and professional competence, while clarifying the negative moderating role of teaching experience. These findings offer several practical implications for the precision-oriented digital empowerment of university PE teachers.

First, professional development systems for PE teachers should emphasize the integrated development of technology, pedagogy, and content. GenAI-related competencies can be embedded into a tiered training framework, in which foundational training focuses on core applications such as action recognition, three-dimensional demonstration, and real-time feedback to ensure operational proficiency. Advanced training may extend to tactical video analysis and physical performance data visualization, enabling teachers to move beyond basic tool use toward higher-level instructional applications. Practice-oriented formats, including lesson study and teaching–research seminars, can further support teachers in iteratively refining their TPACK structures within authentic classroom contexts, thereby promoting the organic integration of technological knowledge and pedagogical strategies.

Second, strengthening teachers’ SRL requires the construction of a data-driven feedback loop supported by GenAI. By leveraging smart teaching platforms, AI-enabled learning dashboards can dynamically present key indicators such as lesson preparation investment, instructional strategy adjustment, classroom interaction, and student feedback. When combined with structured scripts of goal setting, practice, and reflection, these data-driven environments can guide teachers to continuously optimize their SRL strategies. Through repeated cycles of diagnosis, feedback, and improvement, professional competence can be progressively enhanced via sustained self-regulation.

Third, differentiated support strategies should be implemented according to teachers’ career stages. Early-career teachers may benefit most from training that emphasizes advanced GenAI functionalities and research-oriented applications, allowing them to rapidly accumulate digital teaching capital. For mid-career teachers, support should focus on classroom integration and collaborative case development to facilitate the translation of technological tools into stable instructional practices. For senior teachers, professional development initiatives should prioritize technology transfer and embodied cognition applications. Expert demonstration lessons and peer modeling may help reduce barriers associated with the transformation of embodied experience into digitally supported instruction, thereby ensuring that the benefits of GenAI adoption are more evenly distributed across different levels of teaching experience.

## Conclusion

Using three-wave longitudinal data from 558 Chinese university physical education teachers, this study examined the dynamic mechanisms through which generative artificial intelligence adoption contributes to professional competence. The findings indicate that GenAI adoption exerts a significant and sustained positive effect on teachers’ professional competence, demonstrating a cumulative and enduring impact over time. Moreover, self-regulated learning plays a longitudinal mediating role in this relationship, forming a serial pathway from technology adoption to competence development. Importantly, teaching experience negatively moderates this indirect effect, such that early-career teachers benefit more strongly from GenAI adoption through enhanced self-regulation than their more experienced counterparts.

By extending the TPACK framework into a longitudinal perspective and identifying teaching experience as a critical boundary condition, this study advances understanding of how emerging technologies shape teachers’ professional development. These findings provide empirical support for more differentiated and targeted approaches to digital empowerment in physical education.

## Data Availability

The raw data supporting the conclusions of this article will be made available by the authors, without undue reservation.
